# Seroprevalence of severe acute respiratory syndrome coronavirus-2 (SARS-CoV-2) among healthcare providers prior to the vaccine era in an integrated midwestern healthcare system

**DOI:** 10.1017/ash.2022.29

**Published:** 2022-03-23

**Authors:** Elie A. Saade, Xiaochun Zhang, Jaime H Noguez, Lisa M. Stempak, Daniel Tisch, Christine Schmotzer, Robert A. Salata

**Affiliations:** 1 Dvision of Infectious Diseases and HIV Medicine, University Hospitals Cleveland Medical Center, Cleveland, Ohio; 2 Case Western Reserve University, Cleveland, Ohio; 3 Department of Pathology, University Hospitals Cleveland Medical Center, Cleveland, Ohio

## Abstract

We performed severe acute respiratory coronavirus virus 2 (SARS-CoV-2) antinucleocapsid IgG testing on 5,557 healthcare providers and found a seroprevalence of 3.9%. African Americans were more likely to test positive than Whites, and HCWs with household exposure and those working on COVID-19 cohorting units were more likely to test positive than their peers.

The clinical spectrum of coronavirus disease 2019 (COVID-19) is broad, and prognosis is variable; a large proportion of infections remain asymptomatic or minimally symptomatic. Acute infections may be underdiagnosed because of unrecognized exposure, nonspecific clinical presentation, and unequal access to testing. In seroprevalence studies, the presence of IgG antibodies against the severe acute respiratory syndrome coronavirus type 2 (SARS-CoV-2) is used as a surrogate for cumulative incidence of infection in the population at large or specific groups (eg, healthcare providers or other occupations, blood donors, etc).^1,2^

Healthcare workers (HCWs) are considered a high-risk group for exposure to infections by the nature of their frequent, close and prolonged exposure to infected patients and to other HCWs with similarly high risk.^3^ This situation is concerning because HCWs are the backbone of the public health response to any epidemic and they are vital to the functioning of the healthcare system. Additionally, infected HCWs may act as vectors of infections to patients and other HCWs. In parallel, the COVID-19 pandemic has different impacts on subgroups identified by race or ethnicity, occupation, geography, and other characteristics.^4,5^

In this study, we sought to determine the seroprevalence of SARS-CoV-2 among a sample of HCWs, and we explored the effect of occupational and sociodemographic characteristics and self-reported exposure.

## Methods

### Study design and setting

We conducted a cross-sectional voluntary serosurvey at the University Hospitals Health System, a large provider of medical services in northeastern Ohio, comprising a flagship academic center and 11 regional hospitals, with >2,000 beds, 142,000 annual inpatient discharges, and a workforce of 28,000 physicians and employees.

### Participants

All patient-facing HCWs and a sample of non–patient-facing HCWs working at an acute-care facility were invited to participate. Participants were invited by e-mail to review and sign an electronic consent, and reminders were sent to eligible participants. The invitation and reminder e-mails included information on the purpose, design, and procedures of the study, as well as a description of the characteristics, interpretations, and limitations of the serologic tests. Participants were given the opportunity to ask clarifying questions by phone or e-mail to the study team, and they were able to enroll using their organizational credentials to provide consent.

### Data collection

After completing the electronic consent, the participants were directed to an electronic survey hosted on REDCap (Research Electronic Data Capture)^6^ that included demographic and occupational information, medical history, and potential exposure to COVID-19.

### SARS-CoV-2 antibody testing and reporting

Venipuncture and antibody testing were conducted at the University Hospitals Cleveland Medical Center core laboratory which is certified under the Clinical Laboratory Improvement Amendments (CLIA) of 1988 (42 USC 263a) to perform high-complexity testing. The test utilized was the Abbott SARS-CoV-2 IgG assay,^7^ which has been approved by the FDA for use under an emergency use authorization (EUA). Test results were reported qualitatively as negative or positive based on index values using a cutoff of ≥1.4.

### Statistical analysis

We calculated the prevalence of SARS-CoV-2 seropositivity overall and for subgroups of interests; frequencies and proportions are presented. The proportion of seropositive participants in subgroups were compared using a logistic regression model. Possible associations between exposures and seroprevalence were assessed using odds ratios. The variables reported in Table 1 were used to adjust estimates, and the variable “age” was included as a continuous variable. *P* < .05 was regarded as statistically significant.

**Table 1.** Sociodemographic Characteristics of Study Participants and COVID-19 Seropositivity Rate

Factor	Total Sample, No. (%)	Seropositive, No. (%)	Unadjusted Odds Ratio [95% CI]	*P* Value	Adjusted Rate Ratio	*P* Value
All	5,557 (100)	216 (3.9)				
Sex, female	4,504 (82)	180 (4)	1.18 [0.82–1.69]	.38	0.99 [0.67–1.45]	.95
**Age**						
<30 y	1,082 (19.5)	51 (4.7)				
30–39 y	1,710 (30.8)	52 (3.04)				
40–49 y	1,178 (21.2)	47 (4)				
50–59 y	1,032 (18.6)	44 (4.3)				
≥60 y	555 (10)	22 (4)				
**Race**						
White	4,784 (86.1)	173 (3.6)	Reference		Reference	
African Americans	260 (4.7)	23 (8.8)	2.59 [1.64–4.07]	<.01	2.52 [1.57–4.05]	<.01
Asian/Pacific	225 (4)	7 (3.1)	0.86 [0.40–1.84]	.69	1.09 [0.49–2.43]	.83
Hispanic	136 (2.4)	4 (2.9)	0.81 [0.30–2.21]	.68	0.81 [0.29–2.26]	.69
Other/Not available	152 (2.7)	9 (5.9)	1.68 [0.84–3.35]	0.14	1.75 [0.86–3.57]	.12
**Role**						
Nursing	2,048 (36.8)	102 (5)	Reference		Reference	
Physician/APP	1,195 (21.5)	34 (2.8)	0.56 [0.38–0.83]	<.01	0.60 [0.38–0.94]	.03
Other, patient-facing	1,562 (28.1)	52 (3.3)	0.66 [0.47–0.92]	.02	0.71 [0.49–1.04]	.08
Other, non–patient-facing	752 (13.5)	28 (3.7)	0.74 [0.48–1.13]	.16	0.85 [0.53–1.36]	.49
Emergency department	1038 (18.7)	32 (3.1)	0.75 [0.51–1.10]	.14	0.66 [0.44–1.00]	.05
COVID-19 cohorting unit	801 (14.4)	48 (6)	1.74 [1.25–2.42]	<.01	1.66 [1.14–2.41]	<0.01
Collected nasoparyngeal/nasal samples	892 (16)	45 (5)	1.40 [1.00–1.96]	.05	0.98 [0.66–1.46]	.93
**Exposure reported**						
Exposure to patients with COVID-19	2313 (41.6)	106 (4.6)	1.37 [1.04–1.80]	.02	1.29 [0.92–1.81]	.14
Unprotected exposure to patients with COVID-19	731 (13.2)	28 (3.8)	0.98 [0.66–1.47]	.93	0.86 [0.56–1.32]	.50
Exposure to coworker with COVID-19	2,029 (36.5)	94 (4.6)	1.36 [1.03–1.78]	.03	1.21 [0.90–1.63]	.20
Exposure to household member with COVID-19	178 (3.2)	36 (20.2)	7.3 [4.93–10.87]	<.01	7.42 [4.9–11.2]	<.01
Exposure to COVID-19 in the community	409 (7.4)	19 (4.7)	1.22 [0.76–1.98]	.41	0.86 [0.51–1.43]	.55

Note. CI, credible interval; APP, advanced practice provider.

This study was approved by the University Hospitals Ethics Committee (STUDY 20200608).

## Results

**Fig. 1.**
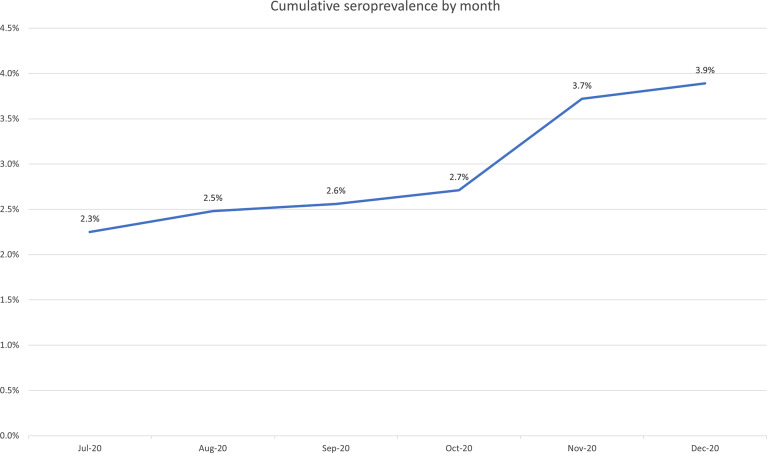
Cumulative monthly seroprevalence during the study period.

In total, 6,278 HCWs enrolled between June 7, 2020, and December 5, 2020, and 5,557 (88.5%) had a blood draw. Monthly cumulative seroprevalence increased from 2.3% in July 2020 to 3.9% by the end of the study period, with a steeper increase in November corresponding to the increase in community incidence of COVID-19 (Fig. 1). Results by demographic and occupational characteristics, and exposure history are provided in Table 1. Notably, Whites comprised the majority of participants and had a seroprevalence rate of 3.6%, followed by African Americans at 8.8%, corresponding to an increased adjusted odds (OR, 2.52; 95% CI, 1.57–4.05; *P* < .01). Nurses represented most participants, and physicians and advanced practice providers (APPs) had statistically significant lower adjusted odds of seropositivity compared to nurses (OR, 0.60; 95% CI, 0.38–0.94; *P* = .03). Participants who reported an exposure to COVID-19 in their household (OR, 7.42; 95% CI, 4.9–11.2; *P* < .01) and those who reported working on a COVID-19 cohort unit (OR, 1.66; 95% CI, 1.14–2.41; *P* < .01) had statistically significantly increased adjusted odds of seropositivity.

## Discussion

During the prevaccination phase of COVID-19, the seroprevalence of SARS-CoV-2 among a sample of HCWs was 3.9%. This rate is slightly higher than the prevalence of 1.5% (95% credible interval [CI], 0.3%–2.9%) measured in a study of a sample of adults in Ohio performed in July 2020.^8^ Similarly, in a study of first responders in northeastern Ohio during May–September 2020, 2.4% were seropositive.^7^ This finding likely reflects the increase of cumulative incidence in the interim.

After adjusting for role, work setting, and self-reported exposure at and outside work, we found significantly higher seroprevalence rates in African Americans (relative to Whites) that persisted. This finding likely reflects the racial and ethnic disparities described with COVID-19, both within and outside the healthcare work setting. A high seroprevalence rate was also observed among those with household exposure, a finding that is in line with other studies. Higher seroprevalence rates were also noted among those who reported working in COVID-19 cohorting units but not among those with other potential work-related exposure, such as working with patients with COVID-19, including with a perceived unprotected exposure, or alongside coworkers diagnosed with COVID-19. This finding might indicate that repetitive and/or longer period of exposure may matter more than a single instance of unprotected exposure. We also noted a lower rate of seropositivity among physicians and APPs, relative to nursing providers. These are diverse groups with widely variable roles and subgroups (by specialty, area of practice, etc) with potentially variable exposure levels, and the lack of granularity in our data did not allow a distinction between different specialties and subgroups.^2–5,9,10^

We acknowledge the limitations of our approach. The use of seroprevalence as a surrogate for the cumulative incidence of past infections is hampered by limitations such as the imperfection and sometimes unknown characteristics of the test used and the decrease in antibody levels with time, possibly below the detection threshold. The timing between infection onset and serology varies and is sometimes unknown, which complicates the interpretation of results. The use of self-reported data is fraught with the risk of recall bias, incomplete reporting, and response inconsistency. Participants self-selected for our study, and despite our efforts to ensure equal opportunity for access, intermediary factors unknown to us might have affected both participation in the study and serology results or other study data. However, there remains general agreement that antibody testing offers valuable information regarding the probable extent of SARS-CoV-2 exposure, the factors associated with exposure and the potential nature and determinants of seropositive status.

In conclusion, although hospital exposure might play a role in acquiring SARS-CoV-2 among HCWs, it is likely that household exposure and sociodemographic characteristics also play a large role in the spread of infection among this group. Healthcare stakeholders should continue to investigate methods to ensure the safety of HCWs and the community at large from existing and emerging infectious agents.
